# The effect of cognitive behavioral therapy on sexual function in reproductive aged women with hypothyroidism: a randomized controlled clinical trial

**DOI:** 10.1186/s12888-023-04870-1

**Published:** 2023-05-23

**Authors:** Azam Sheikh Miri, Mina Iravani, Hatam Boostani, Mahmoud Latifi

**Affiliations:** 1 Department of Midwifery, School of Nursing and Midwifery, Ahvaz Jundishapur University of Medical Sciences, Ahvaz, Iran; 2 Reproductive Health Promotion Research Center, Department of Midwifery, School of Nursing and Midwifery, Ahvaz Jundishapur University of Medical Sciences, Ahvaz, Iran; 3 Department of Psychiatry, School of Medicine, Golestan Hospital, Ahvaz Jundishapur University of Medical Sciences, Ahvaz, Iran; 4 Diabetes Research Center, Department of Statistics and Epidemiology, School of Public Health, Ahvaz Jundishapur University of Medical Sciences, Ahvaz, Iran

**Keywords:** Cognitive behavior therapy, Female sexual dysfunctions, Hypothyroidism

## Abstract

**Background:**

Hypothyroidism is the most common clinical disorder of the thyroid gland which is associated with an increased prevalence of sexual dysfunction even if treated with medication.

**Objective:**

The aim of this study was to determine the effect of cognitive-behavioral therapy (CBT) on sexual function in reproductive-aged women with hypothyroidism.

**Materials and methods:**

This randomized clinical trial was performed on 66 reproductive-aged women with hypothyroidism referring to selected health centers in Izeh, Iran. Data collection tools included demographic information form and Female Sexual Function Index (FSFI). Eligible individuals were randomly assigned to case (n = 33) and control (n = 33) groups using block randomization with the block size of 4. In addition to standard hypothyroidism treatment, the case group received 8 sessions of cognitive-behavioral group therapy, while the control group received only standard treatment.

**Results:**

Before of treatment, there was no significant difference between the mean score of sexual function and its dimensions between the case and control groups (p < 0.05). However, immediately and 4 weeks after completion of treatment, the mean total score of sexual function and its dimensions in the case group increased significantly compared to the control group (p < 0.001).

**Conclusion:**

According to the results of this study, CBT can be effective in improving sexual dysfunction in reproductive-aged women with hypothyroidism. However, before recommending this therapy to women suffering from hypothyroidism, more detailed studies are needed to prove the effectiveness of this intervention, as an adjuvant treatment to the standard pharmacotherapy.

## Introduction

Thyroid is an active hormone gland that forms part of the hypothalamic-pituitary-thyroid axis [1]. Deficiency of thyroid hormone leads to hypothyroidism. The clinical manifestations of this chronic disease are generally nonspecific, so the diagnosis of hypothyroidism is based on the concentration of thyroid-stimulating hormone (TSH) and free thyroxine. Another biochemical condition is subclinical hypothyroidism (SCH), which is characterized by high serum TSH levels but normal levels of free thyroxine and triiodothyronine [2].

With a prevalence of 2 − 10% in different communities [3], hypothyroidism is one of the most common endocrine diseases after diabetes [4–6]. This disease is more common in women and its incidence increases with age [6]. The prevalence of this disease is reported to be approximately 4–8% in the general population and 15–18% in women over 60 [7]. Also, the prevalence of hypothyroidism is higher in women (6 to 8%) than men (3%) [8].

In addition to physical problems such as weight gain, constipation, dry skin, bradycardia, hoarseness, slowed mental processing, and sexual disorders [9], hypothyroidism also causes many psychological symptoms [3].

This disease also increased prevalence of sexual dysfunction in both sexes (men and women). In women, hypothyroidism is associated with disorders of desire, arousal, satisfaction and orgasm, as well as dyspareunia (10–11).

Although, the cause of the relationship between hypothyroidism and sexual dysfunction has not been clearly defined. However, some possible causes including changes in mood and circulating sex hormone levels through peripheral and central pathways, directly and indirectly, may be relevant in this context [12].

Hypothyroidism can be the source of symptoms such as anxiety and stress in patients. Because the level of anxiety and stress in patients with hypothyroidism has been reported to be unfavorable, treatment of the associated mental disorders in these patients, in addition to alternative treatment with levothyroxine, seems necessary [3].

Sexual dysfunction involves a disorder in sexual desire and psychosocial changes that affect the sexual response cycle and cause interpersonal stress and problems [13]. One of the most common approaches in the treatment of sexual dysfunction in women is cognitive-behavioral therapy (CBT) [14]. CBT helps clients develop skills for behavior change, communication with others, problem solving, changing unhelpful beliefs and attitudes, and cognitive reconstruction [15].

Despite different etiologies, people with chronic diseases face similar challenges in managing their disease. These challenges include adjusting their lifestyle, dealing with emotions and psychological responses to chronic illness, coping with disease-related symptoms, and adherence to medication regimens and using a medication regimen. While there are many strategies for self-management or improving self-care activities and optimizing health while living with a chronic illness, CBT is a way that can bring good results for people with chronic physical illnesses. Lukkahatai et al. (2019) in a review study reported the role of CBT in the management of chronic diseases and its effect on controlling symptoms and improving the quality of patients’ life [16].

Halford and Brown also introduced CBT as an adjunctive treatment for people with chronic physical illnesses. In this of patients, items such as psychiatric disorders associated with the illness, difficulty in adapting to the illness, difficulty in compliance with treatment, and problems with the patient’s behaviors will be well managed using cognitive-behavioral therapy [17].

In the meantime, CBT is one of the ways to improve undesirable sexual function. Nezamnia et al. (2020) showed that compared to routine perinatal care counseling based on CBT improves sexual function and sexual self-efficacy of pregnant women [18].

In cognitive therapy, therapists consider the process of cognitive processing to be more important than physiological factors and believe that the discovery of negative autosuggestion is helpful in the successful analysis of sexual problems [19].

Despite the high prevalence of sexual dysfunction in patients with chronic diseases such as hypothyroidism and the importance of treating sexual dysfunction, doctors, health care providers and patients often ignore it [20]. Considering that, standard hypothyroidism treatment alone does not improve libido and sexual function in many women [12], therefore, if the effectiveness of cognitive behavioral therapy as a complementary treatment alongside standard drug treatments is proven, it will help clinicians and health teams to consider this issue in the treatment process of women with hypothyroidism who suffer from sexual disorders.

As far as we know, there is no previous study on this topic. Thus, the present study was conducted to determine the effect of CBT on sexual function in reproductive-aged women suffering from hypothyroidism.

## Materials and methods

### Study design and participants

The present study was a randomized controlled clinical trial performed on 66 reproductive-aged women with hypothyroidism referring to selected health centers (Health Center No. 1, Comprehensive Medical Services Center No. 4) in Izeh, Iran. This research was approved by the Ethics Committee of Ahvaz Jundishapur University of Medical Sciences (Ref. ID: IR.AJUMS.REC.1398.387). This study was also registered in the Iranian registry for clinical trials (Ref. ID: IRCT20200706048030N1, 01/09/2020). The study started on August 18, 2020 and ended on October 19, 2021.

### Inclusion and exclusion criteria

Inclusion criteria: women who were literate, aged between 18 and 45 years, and diagnosed with clinical hypothyroidism by an internal medicine specialist based on TSH levels were eligible to participate in this study. It should be noted that subclinical patients were not included in this study.

Exclusion criteria: women with a history of receiving training based on cognitive-behavioral approach, and a history of known chronic and acute physical and mental illnesses or using psychotropic drugs prescribed by a physician or psychiatrist were excluded from the study.

### Sample size calculation

Based on a study by Atis et al. [9]., assuming the mean ± standard deviation of FSFI total score 32.31 ± 3.50 and 23.92 ± 5.81 in the intervention and control groups, respectively, with 95% confidence (ɑ = 0.05), statistical power of 95% (β = 0.05) and Effect size of 0.95, the sample size was calculated to be 30 women for each group using G-power software. Given the possible 10% attrition rate, 33 women were allocated to each of intervention and control groups.

### Sampling

Sampling started after receiving the code of ethics by the Ethics Committee of Jundishapur University of Medical Sciences in Ahvaz and registering the study in the Clinical Trials Center of Iran. The researcher attended in selected health centers in the Iranian city of Izeh. The women referred to these centers were selected through purposive sampling. The eligible women with diagnosis of clinical hypothyroidism based on laboratory tests were referred to an internal specialist for definitive diagnosis. Then participants were briefed on the objectives of the study, written consent was obtained from them, and they were assured that their information would remain confidential. Finally, 66 women aged 18 to 45 years diagnosed with clinical hypothyroidism and FSFI Score equal to or less than 26.5 who met the inclusion criteria were included in the study.

### Randomization

In this study, participants were randomly assigned into the case (n = 33) and control (n = 33) groups based on block randomization with the block size of 4 and allocation ratio of 1:1 using random sequence generation software.

In order to conceal random allocation, sequentially numbered, sealed opaque envelopes were used. Thus, after performing a random sequence, a number of envelopes with aluminum wrappers (in order to obscure the contents of the envelopes) were prepared in proportion to the research sample size, and each of the random sequences created was recorded on a card. The cards were then placed in envelopes. In order to maintain a random sequence, the envelopes were numbered on their outer surface in the same way. Finally, the lids of the envelopes were glued and the envelopes were placed inside a box. At the time of registration of participants, according to the order of entry of eligible participants into the study, an envelope was opened and the participant was assigned to one group.

Since the study could not be blinded due to the nature of the research, in order to reduce the possibility of bias, the type of intervention was assigned to the participants in the two study groups by a person who was involved in neither sampling nor data analysis. The calculation of the score of the female sexual function questionnaire of the patients was evaluated by a person who was not aware of the study process.

### Intervention

In addition to standard pharmacotherapy, participants in the case group (n = 33) received CBT in 8 sessions, while the control group received only standard pharmacotherapy. After coordination with eligible individuals, treatment sessions were held regularly on a specific day, once a week, for the case group, each sessions lasting from 1.5 to 2 h. CBT was performed by one of the researchers (AS) who had received the necessary training and received the relevant certificate. All treatments were performed under the supervision of an experienced psychiatrist.

A brief description of the sessions is as follows:

Session 1: This session involved introducing the program to the participants, establishing relationships with them, providing a definition and explanation of thyroid disorders and its effect on sexual dysfunction, examining the causes of thyroid disorders, effective factors and history of the problem, examining the medical history of the clients and their physiological condition, examining the relationship between the couple’s interest in each other, the type of their marriage, and the type, frequency and quality of sexual relations between them, conducting sexual interviews, stating the logic, value and importance of treatment, stating the goals of counseling based on CBT, providing a brief explanation about the course and type of treatment, assigning homework.

Session 2: This session was devoted to evaluation of dysfunctional sexual thoughts and beliefs in thyroid disorder. This included the following: Assessment of Illness-related thoughts, emotions, beliefs, behaviors and physical symptoms, evaluating dominant negative sexual attitudes, examining irrational sexual beliefs and explaining them from a scientific point of view, examining sexual preferences and desires of couples and how to express them to each other, reviewing sexual attitudes of women with thyroid disorders, examining the physical and emotional phases of couples during intercourse, assigning homework.

Session 3: This session addressed the cognitive reconstruction and transformation of negative attitudes towards sexual issues in thyroid disorder. This included the following: review of the second session, cognitive reconstruction for psychological adjustment to chronic health problems, cognitive reconstruction for increase the ability to deal with annoying thoughts and negative attitudes related to the disease, cognitive reconstruction for increase the patient’s ability to improve mood, cognitive reconstruction of sexually dysfunctional thoughts in patients with thyroid disorders, homework assignment.

Session 4: This session provided sexual information and knowledge in relation to thyroid disorder. This included the following: introducing the factors contributing to proper sexual dysfunction, getting familiar with sexual organs, physiological functions and their hormones, offering training on sex sensitive points, the benefits of sexual intercourse from a psychological and physical point of view, the role of women in sexual relations, communication skills, assigning homework.

Session 5: This session dealt with training on non-sexual sensate focus. This included the following: reviewing the fourth session, training on body sensate focus, training on developing concentration and attention skills with respect to non-genital organs, examining verbal communication and how couples express their emotions to each other, teaching emotion expression, verbalization of emotional feelings, and sexual self-expression, enhancing intimacy between couples, assigning homework.

Session 6: This session was concerned with training on sexual sensate focus. This included the following: reviewing the fifth session, providing more information about the genitals and sexually sensitive points, increasing self-awareness and sexual self-efficacy, offering training on concentration on sexual organs, attention to excitement and pleasure of the genitals, and sexual fantasies, assigning homework.

Session 7: This session focused on teaching how to have sex in relation to an existing problem. This included the following: reviewing the fifth and sixth sessions, teaching different types of intercourse methods, teaching different types of intercourse methods tailored to the couple’s problem and related techniques, assigning step-by-step homework, G spot point maneuvering, teaching how to reach simultaneous orgasm in accordance with the couple’s sensitive points, assigning homework.

Session 8: This session was dedicated to the assessment of the achievement of therapy goals. This included the following: reviewing the seventh session, evaluating various techniques used by couples, giving feedback on the effectiveness or ineffectiveness of therapy, resolving the existing problems, evaluating the positive results of the therapy program.

In addition, participants were asked to practice the assignments presented in the therapy sessions for 30 min a day, 6 days a week, using the handouts provided.

Because the control group received only standard medical treatment and the routine training provided in health centers, in order to observe ethical considerations and acknowledge their participation, they were given a manual along with a training CD at the end of the intervention.

### Data collection tools

In this study, data were collected using a demographic information form and Female Sexual Function Index (FSFI).

The demographic information form included age, duration of illness, body mass index (BMI), level of education, and occupation.

The Female Sexual Performance Index (FSFI) was developed by Rosen et al. in 2000 [21]. It includes 19 questions that examine women’s sexual function in 6 independent domains of sexual desire (questions 1 and 2), arousal (questions 3, 4, 5 and 6), lubrication (questions 7, 8, 9 and 10), orgasm (questions 11, 12 and 13), satisfaction (questions 14, 15 and 16) and pain (questions 17, 18 and 19). Scoring of the questionnaire was based on a 6-point Likert scale as follows: 0 = No sexual activity, 1 = Almost never or never, 2 = A few times (less than half the time), 3 = Sometimes (about half the time), 4 = Most times (more than half the time), and 5 = Almost always or always. The scores of each domain are obtained by summing up the scores of the items of that domain and multiplying it by the factor number (since in the present questionnaire, the number of items in each domain is not equal, first to balance the domains with each other, scores obtained from the items of each domain are summed up and then multiplied by the factor number). The scores for the questions are 1- desire, 2- arousal, 3- vaginal lubrication, 4- orgasm, 5- pain, and 6- sexual satisfaction (1–5 or 0). A score of zero indicates that the person did not have sexual activity in the past 4 weeks. By summing up the scores of the six domain, the total score of the scale is obtained. A higher score indicates better sexual function. By balancing different domains, the maximum score will be 6 for each domain and 36 for the whole scale. The minimum score will be 1.2 for the domain of ​​sexual desire, 0 for the domains of arousal, lubrication, orgasm and pain, 0.8 for the domain of satisfaction, and 2 for the whole scale. The cut-off points for the whole scale and the domains are: the whole scale: 28, desire: 3.3, arousal: 3.4, lubrication: 3.4, orgasm: 3.4, satisfaction: 3.8, and pain: 3.8. In other words, scores higher than the cut-off point indicate good sexual function [22].

In Rosen et al., the reliability of this questionnaire was evaluated, and the Cronbach’s alpha coefficient for all domains was 0.82 and above, and for the whole scale, it was 0.97 [21]. FSFI is considered the gold standard for assessing women’s sexual function and has been translated and validated in more than 30 countries [23]. In Iran, Mohammadi et al. evaluated the reliability of this scale and its domains by calculating Cronbach’s alpha. The Cronbach’s alpha coefficient obtained for all individuals was calculated to be 0.85, which indicates good reliability of this tool. The validity or reliability of the Persian version showed a significant difference between the case and control groups in terms of the mean score of the whole scale and that of each domain. In this study, the appropriate cut-off point of the whole scale for the diagnosis of sexual dysfunction was 28 [22].

### Outcome assessment

Immediately and 4 weeks after completion of cognitive-behavioral therapy program, the demographic questionnaire and FSFI were completed once again by both case and control groups.

### Statistical analysis

The normal distribution of the data was measured using the Kolmogorov-Smirnov and Shapiro-Wilk tests. Data were analyzed using descriptive statistics (mean, standard deviation, frequency and percentage), independent t-test, chi-square test and repeated measures ANOVA test by SPSS statistical software version 23. P less than 0.05 was considered as statistically significant.

## Results

**Fig. 1:**
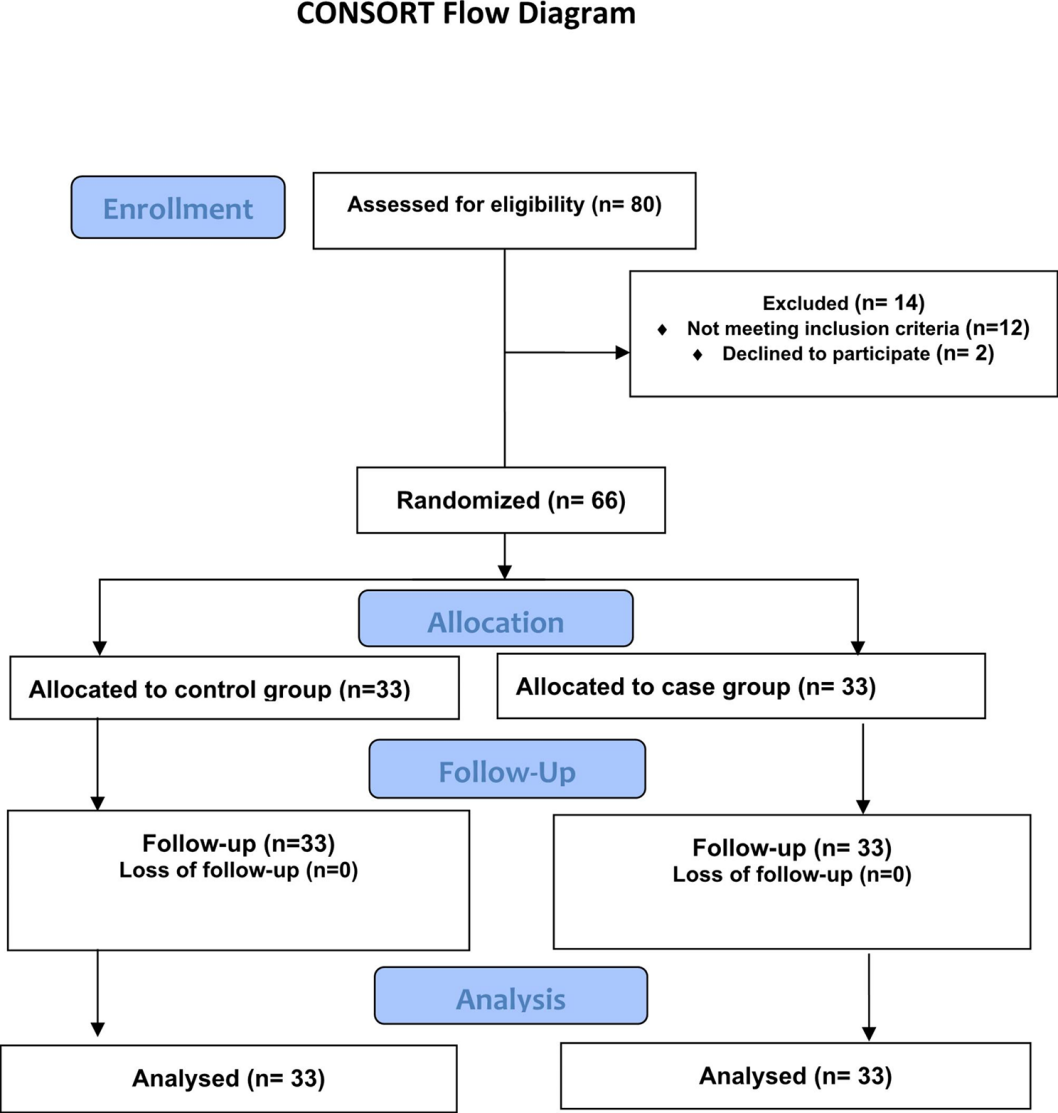
Flowchart of the progress through the phases of the trial

In this study, 66 reproductive-aged women with hypothyroidism were recruited and divided into intervention (n = 33) and control (n = 33) groups. (Fig. 1).

Before the treatment, the difference between the case and control groups in terms of the mean of Age (year), Body Mass index (Kg/m^2^) and duration of illness (year) were not significant (p > 0.05). The mean age of the subjects in the control and case groups was 31.48 ± 6.76 and 31.61 ± 5.01, respectively. Most of the participants in both case and control groups had university education and were housewives (Table 1).

**Table 1:** Comparison of demographic-obstetric characteristics of women with hypothyroidism in case and control groups

Demographic characteristics		Control group (n = 33)	Case group (n = 33)	*P-value
		**Mean ± S**	**Mean ± SD**	
Age (year)		31.48 ± 6.76	31.61 ± 5.01	0.93
Duration of illness (year)		0.36 ± 1.76	0.09 ± 0.52	0.44
Body Mass index (Kg/m^2^)		27.86 ± 4.63	24.55 ± 4.09	0.35
parity		2.18 ± 1.15	2.09 ± 1.04	0.58
		**Frequency (%)**	**Frequency (%)**	**P-value
Education	No high school diploma	11 (33.3)	11 (33.3)	0.85
	High school diploma	7 (21.2)	6 (18.2)	
	University degree	15 (45.5)	16 (48.5)	
Occupation	Housewife	31 (93.9)	29 (87.9)	0.53
	Clerk	2 (6.1)	3 (9.1)	
	Self-employed	0 (0)	1 (3)	
Duration of marriage	Less than one year	10 (30.4)	1 (3)	0.57
	Between 1–5 years	5 (15.2)	7 (21.2)	
	Between 5–10 years	17 (51.5)	13 (39.4)	
	More than 10 years	11 (33.3)	12 (36.4)	

*: Independent t-test, **: Chi-square test

Based on the results this study, it was found that the mean scores of sexual function before, immediately after, and 4 weeks after completion of cognitive-behavioral therapy program were statistically significant in the CBT group (p < 0.05), but there was no significant difference between the mean scores of sexual function in the control group, before, immediately after, and 4 weeks after the start the intervention (p < 0.05).

**Fig. 2:**
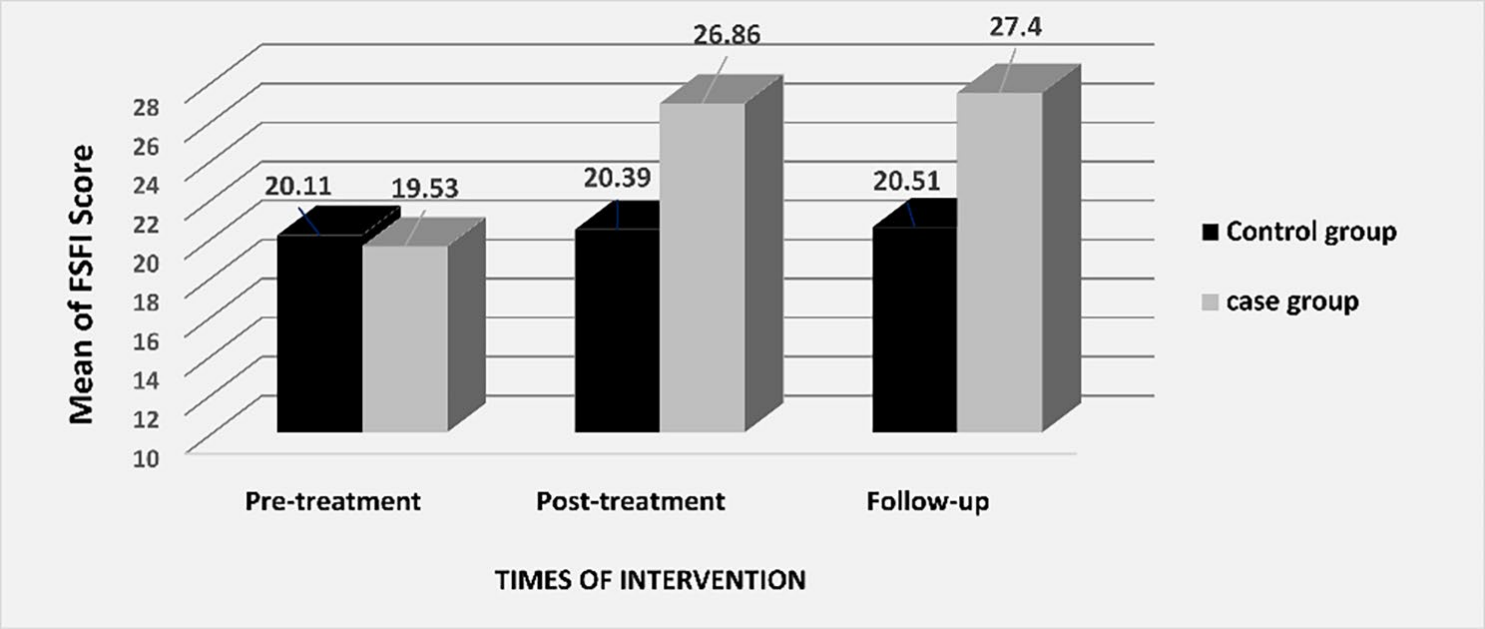
Changes in total sexual function score before and after the treatment in case and control groups *Post-treatment: Immediately after completion treatment; **Follow-up: 4 weeks after completion of treatment

**Fig. 3:**
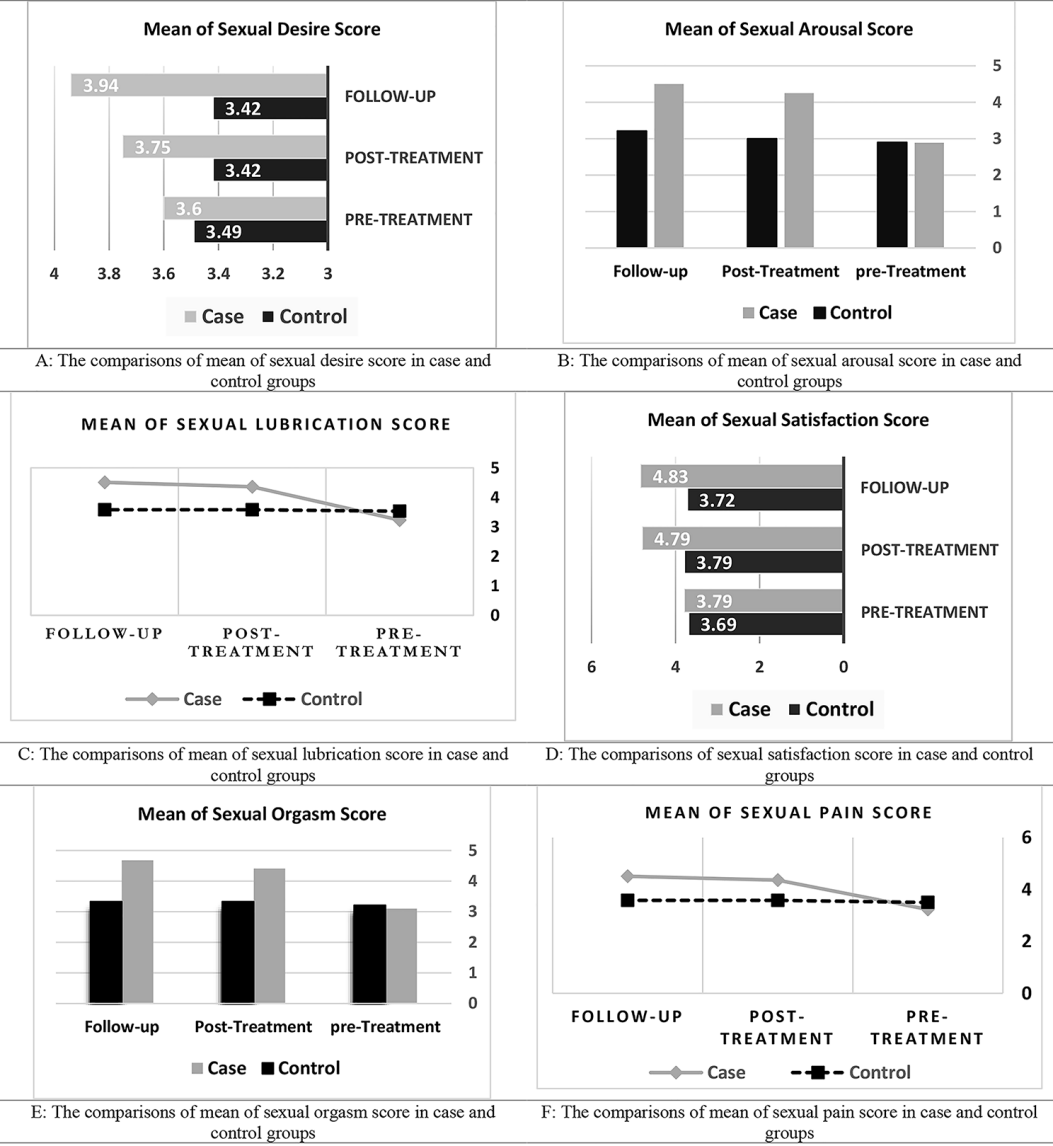
The comparison of Mean of 6 independent domains of the Female Sexual Function Index (FSFI) in case and control groups *Post-treatment: Immediately after completion treatment; **Follow-up: 4 weeks after completion of treatment

Also, the results showed that the difference between the case and control groups in terms of the mean total scores of sexual function and its dimensions (Desire, Arousal, Lubrication, Satisfaction, Orgasm and Pain) before the intervention was not significant (p > 0.05), but immediately and 4 after completion of intervention, this differences were statistically significant (p < 0.05) (Table 2; Figs. 2 and 3).

**Table 2:** Comparison of mean and standard deviation of sexual function and its dimensions in case and control groups before and after cognitive-behavioral therapy

Variable	Time	Control group (n = 33)	Case group (n = 33)	*P-value	&Effect size (%95 CI)
		** Mean ± SD**			
Desire	Pre-Treatment	3.49 ± 0.73	3.6 ± 0.69	0.53	0.155 (-0.328–0.638)
	Post-Treatment#	3.42 ± 0.53	3.75 ± 0.64	0.027	0.562 (0.07–1.054)
	Follow-up$	3.42 ± 0.53	3.94 ± 0.61	0.001	0.91 (0.403–1.417)
**P-value		0.025			
Arousal	Pre-Treatment	2.96 ± 1.29	2.89 ± 1.44	0.83	-0.051 (-0.534–0.431)
	Post-Treatment	3 ± 1.11	4.52 ± 0.65	0.027	1.671 (1.111–2.232)
	Follow-up	3.21 ± 1.05	4.5 ± 0.52	0.001	1.557 (1.006–2.108)
**P-value		0.005			
Lubrication	Pre-Treatment	3.22 ± 1.48	2.89 ± 1.71	0.55	-0.206 (-0.69–0.277)
	Post-Treatment	3.34 ± 1.25	4.13 ± 0.55	0.02	0.818 (0.316–1.32)
	Follow-up	3.52 ± 1.24	4.16 ± 0.47	0.008	0.683 (0.186–1.179)
**P-value		0.13			
Satisfaction	Pre-Treatment	3.69 ± 1.36	3.79 ± 1.29	0.9	0.075 (-0.407–0.558)
	Post-Treatment	3.79 ± 1.3	4.79 ± 0.88	0.001	0.901 (0.394–1.407)
	Follow-up	3.72 ± 1.3	4.83 ± 0.88	0.001	1 (0.488–1.512)
**P-value		0.01			
Orgasm	Pre-Treatment	3.21 ± 1.63	3.1 ± 1.82	0.8	0.064 (-0.419–0.546)
	Post-Treatment	3.33 ± 1.42	4.41 ± 0.91	0.001	0.906 (0.399–1.412)
	Follow-up	3.33 ± 1.42	4.68 ± 0.7	0.001	1.206 (0.681–1.73)
**P-value		0.014			
Pain	Pre-Treatment	3.53 ± 1.79	3.23 ± 1.91	0.51	-0.162 (-0.645–0.321)
	Post-Treatment	3.58 ± 1.67	4.36 ± 1.11	0.021	0.55 (0.059–1.042)
	Follow-up	3.58 ± 1.67	4.51 ± 0.96	0.008	0.683 (0.186–1.179)
**P-value		0.16			
Total score of sexual function	Pre-Treatment	20.11 ± 6.51	19.53 ± 6.89	0.73	-0.087 (-0.569–0.396)
	Post-Treatment	20.39 ± 6.16	26.86 ± 6.79	0.001	0.998 (0.486–1.51)
	Follow-up	20. 51 ± 6.2	27.40 ± 6.70	0.001	1.067 (0.552–1.583)
**P-value		0.005			

#Post-treatment: Immediately after completion treatment; $Follow-up: 4 weeks after completion of treatment

*: Independent t-test, **: Repeated measures analysis of variance; &Effect Size d Cohen

## Discussion

The present study was carried out to evaluate the effect of CBT on sexual function in women with hypothyroidism. According to the findings, CBT improved the six dimensions of female sexual function including desire, arousal, lubrication, orgasm, satisfaction, and pain, as well as the total sexual function score in women with hypothyroidism immediately and 4 weeks after completion of cognitive-behavioral therapy program.

According to Romero-Gómez et al. (2020), compared with controls, the prevalence of sexual dysfunction was higher in women with hypothyroidism despite levothyroxine treatment. Based on their results, hypothyroidism increased the risk of sexual dysfunction, with the most affected domains being desire, orgasm, and pain [12]. Wang et al. (2020) conducted a meta-analysis study which showed that hypothyroidism impairs women’s sexual function to varying degrees [24].

Amiri et al. (2015) reported that in patients with hypothyroidism, the level of anxiety and stress is undesirable, which can be due to hypothyroidism. Therefore, they concluded that in addition to alternative treatment with levothyroxine, treatment of associated psychological problems is essential in these patients [3].Şafak Öztürk et al. (2017) reported that cognitive behavioral therapy is a suitable treatment approach to reduce the level of anxiety and depression and improve sexual performance in women with vaginismus [25].

Mestre-Bach et al. (2022) conducted a review study. They focused on “the psychotherapeutic approaches used in the treatment of female sexual dysfunction disorders” and reported cognitive-behavioral counseling as a successful treatment [26].

Since at the time of writing this manuscript, we found no study on the application of cognitive-behavioral therapy aimed at the treatment of sexual dysfunction in reproductive-aged women with hypothyroidism, we tried to compare the results of the present study with those of studies examining the effect of cognitive-behavioral therapy in individuals with sexual dysfunction without underlying disorders or in patients with other chronic diseases.

The results of Nezamnia et al. (2020) and Ahi et al. (2018) showed that cognitive-behavioral counseling can improve sexual desire in women and lead to increased sexual desire and sexual attraction in them [27–28], which is in line with the results of the present study.

By the same token, Sabetnejad et al. (2016) found that the cognitive-behavioral counseling program together with fluoxetine significantly increases sexual performance in women with obsessive compulsive disorder [29]. Babakhani et al. (2018) found that the cognitive-behavioral counseling program significantly increases sexual performance in women with sexual function disorder [30].

Ziaee et al. (2014) also reported the effect of cognitive-behavioral counseling on vaginal lubrication in women [31]. As far as orgasm was concerned, Babakhani et al. (2018) indicated that the counseling program is effective in increasing orgasm in women and thus increasing sexual function [30]. Shokhmgar et al. (2020) and Tavakolizadeh et al. (2013) also showed that counseling leads to increased marital satisfaction in women with sexual dysfunction (32–33). Moradi et al. (2016) showed that the mean sexual pain in the intervention group decreased significantly compared to the control group [34]. According to Saboula et al. (2015), CBT is an effective treatment to control dyspareunia and reduce the severity of sexual pain [35]. The results of all these studies are consistent with the results of the present study.

Hamid et al. (2012) found that CBT led to increased sexual function in women with vaginismus [19]. In addition, Kaneh et al. (2019) reported the positive effect of cognitive-behavioral counseling program on sexual dysfunction in women [36]. Based on the results of Navidian et al. (2017), counseling in the intervention group could improve sexual function in pregnant women [37]. Therefore, the results of these studies are also in line with those of the present study and confirm the usefulness of interventions based on cognitive-behavioral approach in improving female sexual function.

The hypothyroidism has been shown to be associated with an increased incidence of sexual dysfunction even when normalized with levothyroxine and thyroid stimulating hormone (TSH) [12]. This highlights the importance of considering CBT as a complementary therapy in addition to standard pharmacotherapy.

As mentioned earlier, due to the insufficient studies conducted on this topic, the effect of cognitive-behavioral therapy on improving sexual function and its mechanisms in chronic diseases such as thyroid disorders have not been studied thoroughly yet. Factors such as psychiatric disorders associated with chronic illnesses including health anxiety, coping problems, and difficulty in compliance with treatment, as well as problems with illness behaviors may play a role in the development of sexual dysfunction in patients with chronic diseases CBT has been proposed as a very effective and possibly cost-effective treatment for managing anxiety caused by health problems [38].

Lukkahatai et al. (2019) also pointed to the role of cognitive-behavioral therapy in managing chronic diseases, controlling the symptoms, and improving the quality of life [16].

Cognitive-behavioral therapy is used to treat psychiatric disorders not only in patients with chronic physical illness, but also in those who do not have a psychiatric disorder but have problems with illness-related beliefs, illness behaviors, or illness adaptation [17]. In this regard, various studies have introduced CBT as a complementary and effective therapy in the management and treatment of chronic diseases such as diabetes [39], heart disease [40], sickle cell anemia [41] and cancer [42].

Cognitive-behavioral therapy has been shown to be effective in improving sexual dysfunction in women with diabetes [43]. It has also been found that sexual counseling has a positive effect on the sexual performance of women with diabetes [44]. According to Rezaei et al., CBT can improve some aspects of quality of life in women with hypothyroidism. These include emotional health and the problems associated with it, energy, and general health [45].

Therefore, given the results of studies on the effects of chronic diseases on sexual dysfunction on the one hand and the effect of CBT on the improvement of these disorders on the other, and considering the results of this study, it can be argued that the use of this treatment along with standard treatments can play an important role in promoting sexual function in these patients.

The most important strength of the present study was the novelty of its topic, and it was the first study to investigate the effectiveness of using cognitive-behavioral counseling along with pharmacotherapy to treat sexual dysfunction in women with hypothyroidism. Conducting the treatment sessions under the supervision of an experienced psychiatrist is another strength of this study.

Nevertheless, a limitation that should be taken into account is that this study was conducted during the Covid-19 pandemic, which caused anxiety and stress among the participants for attending the sessions. This problem was minimized by reducing the number of people attending the sessions, providing a completely appropriate space for them, and observing hygienic protocols.

## Conclusion

According to the results of the present study, CBT could improve sexual dysfunction in women with hypothyroidism. Therefore, due to the fact that this treatment is a non-invasive and low-cost intervention, it can be used along with other standard pharmacotherapies. However, more detailed research with a larger sample size is recommended to prove the effectiveness of this treatment.

## Data Availability

The datasets generated and/or analyzed during the current research are not publicly available as individual privacy could be compromised but are available from the corresponding author on reasonable request.

## Abbreviations

CBT: Cognitive-behavioral therapy
TSH: Thyroid-stimulating hormone
SCH: Subclinical hypothyroidism
FSFI: Female sexual function Index
BMI: Body mass index
